# Women exposed to famine in early gestation have increased mortality up to age 76 years

**DOI:** 10.1111/ppe.13131

**Published:** 2024-10-01

**Authors:** Aline Marileen Wiegersma, Tessa J. Roseboom, Susanne R. de Rooij

**Affiliations:** ^1^ Epidemiology and Data Science Amsterdam UMC Location University of Amsterdam Amsterdam The Netherlands; ^2^ Health Behaviors & Chronic Diseases, Aging & Later Life Amsterdam Public Health Research Institute Amsterdam The Netherlands; ^3^ Amsterdam Reproduction and Development Amsterdam The Netherlands; ^4^ Obstetrics and Gynaecology Amsterdam UMC Location University of Amsterdam Amsterdam The Netherlands

**Keywords:** mortality, prenatal famine, prenatal undernutrition, survival

## Abstract

**Background:**

We have previously shown that exposure to famine in early gestation was associated with poorer adult health and, in women, with reduced survival up to age 64.

**Objectives:**

Here, we explore the association between prenatal famine exposure and mortality up to age 76 for men and women separately.

**Methods:**

We studied adult mortality (>18 years) in men (*n* = 989) and women (*n* = 1002) born as term singletons around the time of the 1944–1945 Dutch famine. We compared overall and cause‐specific mortality among men and women exposed to famine in late, mid, or early gestation to that among unexposed persons (born before or conceived after the famine) using Cox regression.

**Results:**

In total, 500 persons (25.1%) had died after age 18. Women exposed to famine in early gestation had higher overall (HR 1.49, 95% CI 1.00, 2.23), cancer (HR 2.17, 95% CI 1.32,3.58) and cardiovascular mortality (HR 2.33, 95% CI 0.91, 5.95) compared to unexposed women. Mortality rates among men were not different between exposure groups.

**Conclusion:**

This study showed that women, but not men, exposed to famine in early gestation had increased overall, cardiovascular and cancer mortality up to age 76. Although prenatal famine exposure affects adult health of both men and women, it seems to only lead to increased mortality among women.

**Synopsis**

**Study question**

Is prenatal famine exposure associated with overall and cause‐specific adult mortality up to age 76 in both men and women?

**What is already known**

We have previously shown that exposure to famine in early gestation was associated with poorer adult health and, in women, with reduced survival up to age 64.

**What this study adds**

The association between prenatal famine exposure and mortality up to age 76 has not been previously studied. This study shows that women, but not men, exposed to famine in early gestation had increased overall, cardiovascular and cancer mortality up to age 76. Although prenatal famine exposure affects the health of both men and women in adulthood, it seems to only lead to increased mortality among women.

## 1 BACKGROUND

Adverse conditions in utero, like prenatal undernutrition or foetal growth restriction, may result in permanent changes in organ structure, cell number and gene expression, which may lead to poorer health throughout life and subsequently reduce adult survival.^1^, ^2^, ^3^

During the winter of 1944–1945, the western part of the Netherlands was struck by an acute period of famine. The circumstances of the Dutch famine were catastrophic, but provided the unique opportunity to study the long‐term consequences of prenatal exposure to undernutrition on morbidity.^4^ Men and women who were prenatally exposed to the Dutch famine had an increased risk for chronic diseases in later life, including coronary heart disease, especially after exposure in early gestation.^4^, ^5^, ^6^, ^7^ Women exposed to famine in early gestation were more centrally obese and had a higher risk for developing breast cancer compared to unexposed women.^8^, ^9^ In a study up to age 64, women, but not men, exposed to famine in early gestation had higher overall, cardiovascular, cancer and breast cancer mortality rates compared to unexposed women.^10^

In recent studies in the Dutch famine birth cohort, we found indications of accelerated brain and cognitive ageing in surviving men who had been exposed to famine in early gestation.^11^, ^12^, ^13^, ^14^ In combination with a higher burden of cardiovascular disease in this group, higher mortality in older age may be expected. In the present study, we revisited the association between prenatal exposure to the Dutch famine and mortality, following‐up the cohort up to the age of 76 years and investigating this association in men and women separately.

## 2 METHODS

### 2.1 Cohort selection

The Dutch famine birth cohort consists of 2414 term singletons born alive in the Wilhelmina Gasthuis hospital in Amsterdam between 1 November 1943 and 28 February 1947. As 160 infants were not registered as newborns in Amsterdam and 263 individuals died or emigrated before the age of 18, 1991 (82%) persons were available for linkage after the age of 18 years. This study complies with the Declaration of Helsinki and was approved by the Institutional Review Board of the Academic Medical Center.

### 2.2 Exposure

The Dutch famine has been described in detail elsewhere.^4^ In accordance with our previous studies, an individual was considered prenatally exposed to famine if the average daily food ration of the mother contained fewer than 1000 kilocalories for at least 13 weeks of gestation.^4^ We considered three 16‐week exposure periods: individuals mainly exposed in late gestation (born between 7 January 1945 and 28 April 1945), mid‐gestation (born between 29 April 1945 and 18 August 1945) or early gestation (born between 19 August 1945 and 8 December 1945).^4^ Both individuals born before (born before 7 January 1945) and conceived after the famine (born after 8 December 1945) acted as a control group.^15^

### 2.3 Outcomes

Causes of death until 11 February 2022 were provided by linking with Statistics Netherlands and using the International Classification of Diseases (ICD) coding system as previously described.^10^, ^16^, ^17^ We categorized the primary cause of death into: cardiovascular diseases (ICD‐10 codes I10‐I15, I20‐I25, I30‐I52 and I60‐I69), cancer (ICD‐10 codes C00‐D48) and other or unknown cause of death.

### 2.4 Statistical analysis

We calculated follow‐up time as the time from birth until death or censoring. Censoring occurred before the end of follow‐up when participants had emigrated, did not consent to sharing their address, had unknown place of residence or could not be linked to the deaths register. In these cases, the date at which the municipal registry had provided information about their status was used. The rate of overall, cardiovascular and cancer mortality among those born before the famine did not differ from the rate among those conceived after the famine and they were combined into one control group. We created Kaplan–Meier survival curves for those exposed to famine during gestation and those unexposed. We used Cox regression to explore the effect of prenatal famine exposure on overall and cause‐specific mortality (>18 years) using age as the time variable. We performed all analyses with STATA (16.1) for men and women together and separately.

### 2.5 Missing data

A censoring time could be constructed for all included participants. In total, 444 individuals (22.7%) were lost to follow‐up during the study.

## 3 RESULTS

Of the 1991 individuals available for follow‐up, 500 (25.1%) individuals had died and 1047 (52.6%) individuals were alive at the end of follow‐up. In total, 444 (22.7%) were lost to follow‐up. In total, 683 (34.3%) participants were considered exposed to famine in utero of whom 251 were exposed to famine in late gestation, 248 in mid gestation and 184 in early gestation.

Individuals exposed to famine in late or mid gestation were lighter at birth and had smaller head circumference compared to those unexposed to famine, whereas those exposed to famine in early gestation had a slightly higher birth weight (Table 1).

**TABLE 1:** Characteristics by prenatal exposure to the Dutch famine.

	*n*	Born before	Exposure to famine			Conceived after	Total
			In late gestation	In mid gestation	In early gestation		
*General characteristics*							
Number at risk at age 18 years (%)	1991	626	251	248	184	682	1991
Total accumulated observation time	1991	41,427	17,330	16,473	12,132	43,748	131,111
Number of women (%)	1991	305 (48.7)	138 (55.0)	132 (53.2)	99 (53.8)	328 (48.1)	1002 (50.3)
Maternal age at birth (SD)	1991	28.7 (6.3)	30.4 (6.8)	28.3 (6.3)	27.6 (6.1)	28.0 (6.4)	28.5 (6.4)
Birthweight (SD)	1991	3382 (457)	3163 (446)	3234 (435)	3492 (474)	3425 (486)	3361 (475)
Head circumference (SD)	1976	32.9 (1.6)	32.3 (1.7)	32.1 (1.4)	32.9 (1.4)	33.2 (1.6)	32.8 (1.6)

*Note*: Numbers represent frequencies (%) or means (SD).

**FIGURE 1:**
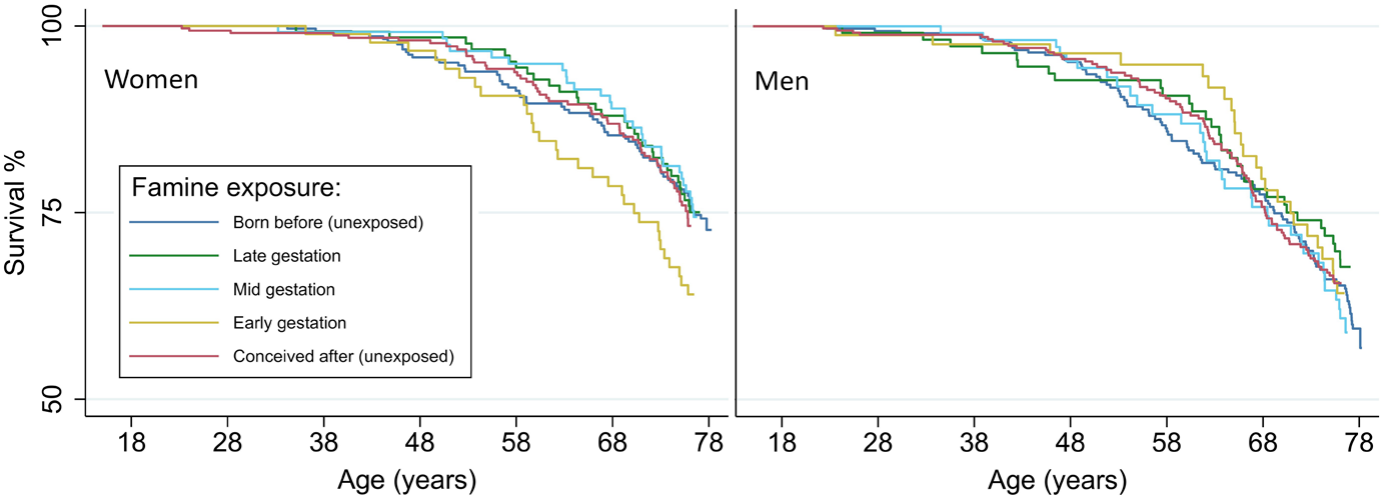
Kaplan–Meier survival curves for men and women born before the famine, exposed mainly in late, mid or early gestation and those conceived after the famine.

When analysing both sexes combined, there were no differences in overall, cancer and cardiovascular mortality between those exposed to the famine during gestation and those who had not been exposed (Table 2; Figure 1). Those exposed to famine in late gestation had died less often from other or unknown causes of death compared to unexposed individuals (HR 0.51, 95%CI 0.30, 0.87).

**TABLE 2:** Overall, cardiovascular and cancer mortality expressed as HRs (95% CI), for women and men between 18 and 76 years for those exposed to famine in late, mid or early gestation compared to those unexposed (born before and conceived after the famine).

Cause of mortality	*n*	Born before	Prenatal famine exposure			Conceived after
			Late gestation	Mid gestation	Early gestation	
Overall adult						
*N* (%)	500 (25.1)	166 (26.5)	63 (25.1)	64 (25.8)	54 (29.3)	153 (22.4)
Hazard ratio (95% CI)		1.00 (Reference)	0.85 (0.65–1.12)	0.98 (0.75–1.28)	1.17 (0.88–1.56)	1.00 (Reference)
Cardiovascular						
*N* (%)	97 (4.9)	28 (4.5)	12 (4.8)	13 (5.2)	13 (7.1)	31 (4.5)
Hazard ratio (95% CI)		1.00 (Reference)	0.85 (0.46–1.59)	1.05 (0.57–1.91)	1.50 (0.82–2.74)	1.00 (Reference)
Cancer						
*N* (%)	223 (11.2)	73 (11.7)	36 (14.3)	24 (9.7)	29 (15.8)	61 (8.9)
Hazard ratio (95% CI)		1.00 (Reference)	1.17 (0.81–1.70)	0.87 (0.56–1.35)	1.48 (0.99–2.21)	1.00 (Reference)
Other or unknown cause						
*N* (%)	180 (9.0)	65 (10.4)	15 (6.0)	27 (10.9)	12 (6.5)	61 (8.9)
Hazard ratio (95% CI)		1.00 (Reference)	0.51 (0.30–0.87)	1.07 (0.70–1.62)	0.67 (0.37–1.22)	1.00 (Reference)
*Women*						
Overall adult						
*N* (%)	215 (21.5)	64 (21.0)	31 (22.5)	30 (22.7)	30 (30.3)	60 (18.3)
Hazard ratio (95% CI)		1.00 (Reference)	0.92 (0.62–1.36)	0.93 (0.62–1.39)	1.49 (1.00–2.23)	1.00 (Reference)
Cardiovascular						
*N* (%)	31 (3.1)	12 (3.9)	3 (2.2)	6 (4.6)	6 (6.1)	4 (1.2)
Hazard ratio (95% CI)		1.00 (Reference)	0.68 (0.20–2.32)	1.41 (0.55–3.62)	2.33 (0.91–5.95)	1.00 (Reference)
Cancer						
*N* (%)	114 (11.4)	29 (9.5)	21 (15.2)	12 (9.1)	21 (21.2)	31 (9.5)
Hazard ratio (95% CI)		1.00 (Reference)	1.30 (0.79–2.14)	0.78 (0.42–1.45)	2.17 (1.32–3.58)	1.00 (Reference)
Other or unknown cause						
*N* (%)	70 (7.0)	23 (7.5)	7 (5.1)	12 (9.1)	3 (3.0)	25 (7.6)
Hazard ratio (95% CI)		1.00 (Reference)	0.53 (0.24–1.17)	0.95 (0.50–1.79)	0.38 (0.12–1.22)	1.00 (Reference)
*Men*						
Overall adult						
*N* (%)	285 (28.8)	102 (31.8)	32 (28.3)	34 (29.3)	24 (28.2)	93 (26.3)
Hazard ratio (95% CI)		1.00 (Reference)	0.85 (0.58–1.23)	1.12 (0.78–1.61)	0.95 (0.62–1.46)	1.00 (Reference)
Cardiovascular						
*N* (%)	66 (6.7)	16 (5.0)	9 (8.0)	7 (6.0)	7 (8.2)	27 (7.6)
Hazard ratio (95% CI)		1.00 (Reference)	1.05 (0.51–2.15)	1.00 (0.45–2.22)	1.22 (0.55–2.72)	1.00 (Reference)
Cancer						
*N* (%)	109 (11.0)	44 (13.7)	15 (13.3)	12 (10.3)	8 (9.4)	30 (8.5)
Hazard ratio (95% CI)		1.00 (Reference)	1.07 (0.61–1.87)	1.04 (0.56–1.91)	0.82 (0.39–1.70)	1.00 (Reference)
Other or unknown cause						
*N* (%)	110 (11.1)	42 (13.1)	8 (7.1)	15 (12.9)	9 (10.6)	36 (10.2)
Hazard ratio (95% CI)		1.00 (Reference)	0.53 (0.25–1.09)	1.27 (0.73–2.22)	0.94 (0.47–1.88)	1.00 (Reference)

Among men, we did not observe differences in overall mortality after prenatal exposure to famine compared to unexposed men, nor any differences for cause‐specific mortality (Table 2; Figure 1). Women exposed to famine in early gestation had higher overall adult mortality (HR 1.49, 95% CI 1.00, 2.23), cancer (HR 2.17, 95% CI 1.32, 3.58) and cardiovascular mortality rates (HR 2.33, 95% CI 0.91, 5.95) compared to unexposed women. Although the results for cardiovascular mortality lack precision.

## 4 COMMENT

### 4.1 Principal findings

In line with our previous observations on mortality up to age 64, we observed increased overall, cardiovascular, and cancer mortality among wom.

### 4.2 Strengths of the study

Strengths of the current study include the semi‐experimental setting, the completeness of the birth records allowing us to follow the cohort from before birth until more than seven decades later, and the ascertainment of cause of death through national registry data. The Dutch famine was severe, relatively brief and exposure was relatively independent from other factors, thereby limiting the risk of confounding bias.

### 4.3 Limitations of the data

The current study has limitations. First, we were not able to follow 22.3% of included individuals until the end of follow‐up due to emigration, unknown place of residence or not being matched to the deaths register. Although it remains possible that censoring has caused bias, censoring events typically occurred before age 50 and we assume they were mostly independent from mortality risk. Secondly, famine exposure was based on date of birth, which may have led to misclassification and would result in an underestimation of a true effect.

### 4.4 Interpretation

In our current analyses, women exposed to famine in early gestation had the highest mortality rates of all male and female groups. Cancer was the largest contributor to excess mortality in women exposed to famine in early gestation. These findings are in line with the greater risk for developing cancer and higher cancer mortality observed in both men and women exposed to the 1959–1961 Great Chinese famine during gestation.^18^, ^19^, ^20^ Changes in the epigenetic regulation of genes, for instance genes related to cell division and apoptosis could be a pathway via which prenatal undernutrition may increase cancer risk.^21^, ^22^, ^23^ Such epigenetic effects have been demonstrated in a different cohort investigating exposure to the Dutch famine, mainly after exposure in early gestation.^24^

In contrast to women, men exposed to famine in early gestation did not have increased mortality compared to unexposed men up to age 76. This was surprising as, similar to women, men exposed to famine in early gestation did have increased rates of chronic diseases and more signs of accelerated ageing compared to unexposed men.^4^, ^5^, ^6^, ^7^

Different factors may explain why prenatal famine exposure has not resulted in increased mortality in men (yet). First, there are sex differences in prenatal development.^25^, ^26^ During the famine, relatively fewer boys were born alive, possibly due to better survival chances of female foetuses.^27^, ^28^ Due to selective mortality of potentially weaker male foetuses after famine exposure, men born alive after prenatal famine exposure may have had a relatively better survival prognosis. In addition, we have previously observed that the consequences of prenatal undernutrition on morbidity differ between the sexes. Both differences in prenatal development as well as differences in adult physiology between men and women may explain the differences in adult disease and mortality risk after prenatal famine exposure. Finally, cardiovascular diseases manifest themselves differently in men and women and due to gender bias in research the effectiveness of treatments is generally better for men.^29^, ^30^ Gender inequities in diagnosis and treatment may have resulted in relatively lower survival of women with cardiovascular disease.

## 5 CONCLUSIONS

This study shows that women – but not men – exposed to famine in early gestation had increased overall, cardiovascular and cancer mortality up to age 76. Although prenatal famine exposure affects the health of both men and women in adulthood, it seems to only lead to increased mortality among women.

## AUTHOR CONTRIBUTIONS

All authors designed the study. The results are based on calculations by AMW using non‐public microdata from Statistics Netherlands. AMW drafted the manuscript and all authors critically reviewed and revised the manuscript.

## FUNDING INFORMATION

This work was supported by a Dutch Research Council (NWO) Aspasia grant (grant number 015014039) awarded to S. R. de Rooij and by the European Commission Horizon 2020, project LongITools (grant number 874739). The funding organizations had no role in any part of this study.

## CONFLICT OF INTEREST STATEMENT

The authors have no relevant financial or non‐financial interests to disclose.

## SOCIAL MEDIA QUOTE

This study shows that women, but not men, exposed to famine in early gestation had increased overall, cardiovascular and cancer mortality up to age 76. Although prenatal famine exposure affects adult health of both men and women, it seems to only lead to increased mortality among women.

## Data Availability

The data underlying the results presented in the study are based on the Dutch famine birth cohort and non‐public data from Statistics Netherlands. Data can be requested from Statistics Netherlands in combination with data that can be made available by the corresponding author upon reasonable request.
